# Outcomes in a digital weight management intervention with one-on-one health coaching

**DOI:** 10.1371/journal.pone.0232221

**Published:** 2020-04-30

**Authors:** Jordan M. Silberman, Manpreet Kaur, Jakob Sletteland, Aarathi Venkatesan

**Affiliations:** Vida Health, San Francisco, CA, United States of America; McMaster University, CANADA

## Abstract

**Background:**

Some guidelines state that in-person weight management interventions are more efficacious than those delivered digitally. However, digital programs are more scalable and accessible. We hypothesized that one-on-one health coaching via app-based video chat would simulate an in-person experience and help achieve outcomes comparable to those of in-person interventions.

**Methods:**

A 12-month digital weight management intervention was provided to overweight or obese adults recruited from a large technology company. One-on-one health coaching sessions were offered during a 24-week intensive phase as well as subsequent maintenance phase. Focused on sustainable changes in activity and diet, the intervention incorporates SMART goals, in-app food and activity logs, Fitbit integration, as well as optional sleep and stress modules. Self-Determination Theory and the Transtheoretical Model are incorporated to drive behavior change. Multilevel mixed-effects models were used to analyze weight changes retrospectively.

**Results:**

Six hundred eighty-three participants reported 29,051 weights. At 12 months, mean percent changes in body weight were -7.2% and -7.6% for overweight and obese groups, respectively. A weight change of -5% is commonly targeted for in-person weight management interventions. Observed weight loss exceeded this target by 2.2% (95% CI, 0.7% to 3.8%; *P* < .01) for the overweight group and 2.6% (95% CI, 1.4% to 3.9%; *P* < .01) for the obese group.

**Conclusions:**

Further research is needed with randomization to in-person or digital interventions. Though limited by an observational, retrospective design, preliminary results suggest that some digital weight management programs with one-on-one coaching may achieve outcomes comparable to those of robust, in-person interventions.

## Introduction

Obesity continues to show epidemic prevalence levels in the United States; estimates using non-self-reported data from 2015–16 suggest that 40% of U.S. adults are living with obesity, while 32% are overweight [1,2]. Progressing gradually over a period of years, obesity is a pernicious condition associated with increased risk for more than 50 other diseases, including type 2 diabetes, hypertension, dyslipidemia, obstructive sleep apnea, depression, gastroesophageal reflux disease (GERD), nonalcoholic fatty liver disease, osteoarthritis [3,4], and at least 13 types of cancer [5]. Cost estimates vary, but a recent meta-analysis incorporating a variety of findings estimated that over $140 billion is spent annually in the U.S. on direct medical expenses attributable to obesity [6]. The combination of epidemic prevalence, profound health impact, and exacerbation of escalating healthcare costs makes obesity a critical public health challenge. Innovative, scalable intervention strategies are needed to treat and prevent obesity in the United States population.

Evidence-based digital health interventions (DHIs) are one tool that has received increased attention recently as a population health management strategy to help control the obesity epidemic [7]. Often delivered through smartphone apps, web platforms, or both, DHIs can incorporate a variety of program components that may support patients' efforts to achieve sustained behavior changes and manage chronic conditions. DHI components can include food logs, physical activity tracking tools, evidence-based goal setting exercises, educational activities, and integration with devices (activity trackers, sleep trackers, glucometers, etc.), to name a few. A flexible modality, DHIs can be tailored to meet the needs of diverse populations, and can utilize adaptive algorithms to adjust protocols for each patient's unique needs.

In addition, DHIs can incorporate interaction with a health professional, such as a health coach. Patients can interact with this professional using audio or video chat tools as well as text messaging functionality. This can be useful for providing accountability, addressing barriers to health behavior change, identifying and cultivating intrinsic motivators, offering one-on-one health education, and filling the gaps between in-person provider visits.

Recent guidelines [8,9] recommend in-person lifestyle modification programs, when possible, over digital health interventions or other remotely-delivered programs. These guidelines state that in-person weight management interventions are more efficacious, on average, than those delivered remotely. One study [10] found favorable weight loss outcomes for an in-person obesity management intervention relative to a similar, Internet-based program. Meta-analytic evidence, moreover, has suggested better weight outcomes for in-person relative to Internet-delivered interventions [11].

Guidelines [8,9] and evidence [10,12] do suggest, however, that remotely delivered programs can provide clinically meaningful weight loss benefits, and may be worth considering for some patients. Digital health interventions do have important advantages. They may be more accessible for some patients, and provide a valuable treatment option to patients for whom weight management programs would otherwise be unavailable [10]. In addition, DHIs are usually more cost-effective and thus scalable [13]. Ideally, weight management interventions would achieve both the efficacy of robust in-person interventions and the scalability of DHIs. By incorporating one-on-one, app-delivered, video chat-based health coaching into an evidence-based weight management DHI, it may be possible to simulate an in-person experience, and achieve outcomes comparable to those of in-person weight management programs.

The aim of the present study was to investigate changes in weight outcomes among adult participants in a weight management DHI incorporating one-on-one health coaching. Our primary objective was to compare weight outcomes observed in the DHI to weight loss benchmarks often targeted by in-person interventions. A secondary objective was to investigate outcomes differences across demographic groups. We hypothesized that adults who participate in a DHI with one-on-one health coaching would show a mean 12-month weight reduction of 5% or greater, an amount commonly targeted by in-person weight management interventions. Moreover, we hypothesized that weight reductions of at least 5% would be observed across genders and adult age groups.

## Materials and methods

### Design

A retrospective, observational design was utilized to investigate weight changes in a 12-month digital weight management intervention with one-on-one health coaching. An independent institutional review board (Western Institutional Review Board) approved this study. The informed consent requirement was waived because data were fully anonymized before they were accessed for retrospective analyses. Moreover, results are reported only at aggregated levels of analysis.

### Study sample and recruitment

Participants were recruited from a large technology company. Headquartered in San Jose, CA, the company sponsors this digital health intervention for adult employees. Program personnel recruited participants at employer-sponsored, on-site social events and health screenings. Signage, fliers, and email announcements were utilized to promote the program.

Program personnel, as well as flyers and signage, provided information about a special enrollment website. The site presented general information about the program; those interested could enter their mobile phone number through this website. An automated SMS message was then sent to the number entered, containing a download link through which prospective participants could download the vendor's smartphone app. Alternatively, the website provided instructions for downloading the app directly from the App Store (iOS) or Google Play Store (Android).

After installing the app, participants were presented with a series of in-app onboarding forms, through which they provided their email address, demographic information, height, self-reported weight, self-reported health conditions, and a vendor-provided code to confirm eligibility. In addition to requiring that participants own a smartphone, non-pregnant adults (age 18 or older) were considered eligible if they could read and write fluently in English, had a BMI (based on self-reported height and weight) of 25 or greater, and reported no health conditions that could make light-to-moderate physical activity unsafe.

### Health coaches

Health coaches included registered dieticians, certified diabetes educators, certified health education specialists, certified personal trainers, and National Board Certified health & wellness coaches. All coaches are health professionals with significant experience in cardiometabolic health and behavior change. Minimum qualifications for health coaches included a four-year undergraduate degree in a health-related field (eg, nutrition or exercise physiology), a health coaching certification from an accredited organization, as well as 5 years of health coaching experience. Coaches receive extensive training from the program vendor to ensure clinical quality, protocol fidelity, and adherence to HIPAA guidelines for confidentiality. All coaches receive additional training in evidence-based best practices for autonomy support, health behavior change, and weight management.

### Intervention

The intervention was provided by Vida Health (San Francisco, CA), a digital therapeutics company. Delivered largely through a HIPAA-compliant smartphone app (iOS or Android), the intervention incorporates SMART goals, in-app food and activity logs, Fitbit^™^ integration, as well as optional sleep and stress modules. App-delivered content utilizes evidence-based approaches to health behavior change, incorporating goal setting, self-monitoring, self-efficacy building, and problem-solving strategies for overcoming barriers to change. During the initial, intensive program phase, 16 one-on-one health coaching sessions are offered, through video chat in the Vida app (audio-only calls are used if preferred). The first 8 sessions are weekly, while the next 8 sessions occur every other week. In the subsequent maintenance phase, coaching sessions are offered once per month. Throughout both intensive and maintenance phases, participants can use HIPAA-compliant, in-app text messaging to ask their coach questions. Health coaches often use this tool to send reminders, encouragement, or congratulations between coaching sessions.

Each participant works with their coach to choose and track concrete behavior change goals that are likely to impact energy balance. Proprietary machine learning algorithms are used to tailor program content selection and sequencing. The intervention incorporates principles of Self Determination Theory [14], with a focus on establishing sustained changes in behavior.

### Theoretical foundations of the digital health intervention

Health coaching has a modest but growing evidence base as an intervention to support health behavior change. It has been applied successfully in domains including weight management, physical activity promotion, and medication adherence [15–17].

Applying principles of Self-Determination Theory (SDT), the coaching and digital health intervention described herein provides in-app exercises designed to help participants identify key motivators for health behavior change. The SDT literature suggests that intrinsic and identified motivation, as well as other classes of motivation, may promote behavior change more durably than types of motivation that are closer to the extrinsic end of the intrinsic-extrinsic spectrum [14,18]. Intrinsic motivation indicates enjoyment of a behavior itself, irrespective of the behavior's sequelae. Identified motivation arises when someone believes there is personal value in benefits that may be obtained through a behavior. Because the DHI targets long-term maintenance of behavior changes, intrinsic and identified motivation are two areas of focus.

In-app exercises are designed to help each participant identify and amplify intrinsic and identified motivations for health behaviors change. For example, participants are asked to share reasons why they may wish to increase physical activity. In addition to entering their own unique motivators, they are presented with a list of common intrinsic and identified motivations for increasing physical activity, and asked to select any they endorse. Intrinsic motivations for physical activity may include enjoying the outdoors while hiking, enjoying music played during a workout class, etc. Identified motivations could include a desire to stay healthy for as long as possible, to watch grandchildren grow up. The participant's self-reported motivations are shared with coaches. Health coaches can subsequently reinforce the participant's own motivations, and gently remind participants of these motivations at opportune times.

In addition, health coaches incorporate an autonomy-supportive, SDT-concordant approach. Employing techniques such as active listening, empathy, and non-judgmental positive regard, coaches develop rapport with participants and support each participant's perceived autonomy throughout the behavior change process. These techniques may help coaches establish a more collaborative partnership with the participant, which serves as a foundation for subsequent goal setting.

Motivational Interviewing (MI)—a theoretical framework that overlaps with SDT [19]—is also incorporated into the health coaching approach. Coaches employ MI techniques such as open-ended questioning, reflecting, and summarizing participant statements to develop an understanding of each participant's values. Goals of this MI-informed coaching practice include identifying the participant-determined focus for change, as well as exploring and resolving any ambivalence around health behavior change. Health coaches partner with participants in an iterative process of goal setting, reviewing and reassessing, providing accountability, and supporting healthy behaviors.

### Body weight outcomes

Body weight was the primary outcome measure in this retrospective study. Participants were encouraged to measure weight at least once per week, ideally in the morning after the first urination of the day, using a bathroom scale placed on a hard floor.

A "device agnostic" platform, the smartphone app can receive data from a variety of connected bathroom scale products. Manufactured by companies such as BodyTrace^™^ and Withings,^™^ these digital scales connect wirelessly to the smartphone app, and transmit data automatically. All data transfers are secure and HIPAA compliant. Alternatively, participants who did not have a connected scale could utilize a traditional scale, and enter weight readings manually into the app. While 65% of weight observations were transmitted automatically, 35% were entered manually. A sensitivity analysis (described below) investigated whether results differed across measurement methods.

### Engagement outcomes

Secondary outcomes included engagement variables that may reflect the degree of participation in components of the digital health intervention. The following engagement variables were investigated: frequency of health coaching sessions, number of messages sent to the coach, counts of unique app opens, as well as the number of program lessons opened and completed. Participant viewing of "content cards" (brief pieces of content on various health topics, sent at the coach's discretion) was also measured. Frequencies were assessed across four types of self-monitoring behaviors: manual tracking (eg, meal logging), automated tracking (eg, passive step count monitoring using a FitBit), tracking of boolean metrics (custom behavioral goals, like walking 20 minutes per day), and viewing summaries of health-related metrics (eg, the last five weights logged). Weigh-in counts—the number of weight observations recorded per participant—were also included as a measure of engagement.

Weigh-in counts were derived from body weight data, which were collected as described above. Data for all other engagement variables were collected and transmitted to study personnel automatically, by the Vida Health app.

The intervention protocol called for coaching sessions far more frequently during the initial, intensive phase; session frequency decreased to a monthly cadence subsequently. Session counts were of greatest interest during the intensive phase and were therefore the focus of analyses. Similarly, counts of lesson opens and completions were analyzed for the intensive program phase.

### Statistical analysis

Hypotheses were tested using multilevel mixed-effects modeling, which accommodated heterogeneity across participants in the frequency and timing of weight observations, as well as the nested structure of available data (weight observations nested within participants; participants nested within coaches) [20]. Fixed effects specified in our primary model were program time in weeks, baseline BMI category (overweight or obese), as well as the time ✕ BMI category interaction. Overweight was defined as a BMI of at least 25 but less than 30, while obese was defined as a BMI of 30 or greater.

Obesity prevalence is a concern across all age groups and both genders [1]; a subsequent model was therefore estimated to investigate results by demographic categories. Three age groups were specified: 18–39 years of age, 40–59 years of age, and 60 years of age or older. Main effects were included for age group and gender, as well as their interactions with time and each other. Both models included random intercepts at coach and participant levels, and random slopes for time at the participant level. Coach-level random slopes for time were not significant (details below) and were therefore excluded. Models were estimated using data from all participants with 2 or more weight observations available.

Models were used to test the null hypothesis of zero weight change relative to baseline; in addition, observed weight changes were compared to those targeted by in-person weight management interventions. A 5% weight reduction relative to baseline is likely the most widely-used clinical target for in-person weight management programs. This target, for example, is used in CDC-recognized diabetes prevention programs [21]. Weight reduction of 5% is often targeted because it has been shown to produce clinically meaningful benefits, including reductions in hemoglobin A1c, blood pressure, and LDL [9,22]. Differences between observed 12-month weight changes and this 5% weight reduction benchmark were investigated across BMI categories and demographic groups.

Engagement variables were significantly right skewed (all *P*'s < .01; testing procedure detailed elsewhere [23]) and were therefore right Winsorized at the 99th percentile. This retains "power users" without allowing any single participant to have an inordinate impact on results. Engagement variables were averaged across program weeks, within participants. A third model was estimated, with weight predicted from program time, each of the aforementioned engagement variables, and interactions between time and each engagement variable.

Some weights were measured using automated data transmission from a connected digital scale. In other cases, weight was measured using a traditional scale, with weight values entered manually by the participant. Weight values collected during onboarding, moreover, were self-reported. In a sensitivity analysis, full model results were compared to results observed when manually-entered weights—which may be less reliable—were excluded.

To assess risk of attrition bias, 9-month weights were compared across 2 groups: participants who had weight observations available at or after 12 months, and those who did not. If post-9-month weights differ systematically as a function of post-9-month attrition, then we would expect 9-month weights to differ across these groups. This analysis makes the reasonable assumption that 9- and 12-month weights correlate within participants.

Wald asymptotic confidence intervals and *P* values were utilized, an appropriate method for this large sample [24]. Heterogeneities across participants in baseline weights and rates of change were tested with likelihood ratio χ2 tests [20]. Stata/IC 14.0 (StataCorp, College Station, TX) was used for all analyses, with two-sided *P*'s < .05 defined as statistically significant.

## Results

Of 1,253 adults who completed screening and enrolled in the Vida weight management intervention, 84 (6.7%) transitioned to other Vida programs, while 457 (36.5%) had only one weight observation available and were therefore excluded. Another 29 participants (2.3%) were excluded due to other missing data (eg, missing values for height and thus BMI). The remaining 683 (54.5%) adults had 2 or more weight observations available and were therefore included in analyses. A total of 29,051 weights observed across the 12-month study period were analyzed. Participants reported a mean (SD) of 42.5 (90.4) weight observations.

Fifty-four health coaches worked with study participants. On average, each coach worked with 12.6 participants (SD, 12.1). Mean age at baseline was 42.0 (SD, 10.4) and 44.1% were female (Table 1). At baseline, 53.1% (*n* = 363) were overweight (25 ≤ BMI < 30), while 46.9% (*n* = 320) were obese (BMI ≥ 30). Race and ethnicity data were not available.

**Table 1:** Baseline characteristics.

Characteristic	Treatment Group (*N* = 683)
Age in Years, mean (SD)	42.0 (10.4)
Sex, No. (%)	
Female	301 (44.1%)
Male	382 (55.9%)
BMI category, No. (%)	
Overweight (25 ≤ BMI < 30)	363 (53.1%)
Obese (BMI ≥ 30)	320 (46.9%)
BMI,^a^ mean (SD)	30.9 (5.1)
Weight, mean (SD), pounds	202.7 (40.5)
Height, mean (SD), inches	67.6 (3.9)

Abbreviations: BMI, body mass index.

^a^Calculated as kilograms divided by height in meters squared.

### Body weight outcomes

**Fig 1:**
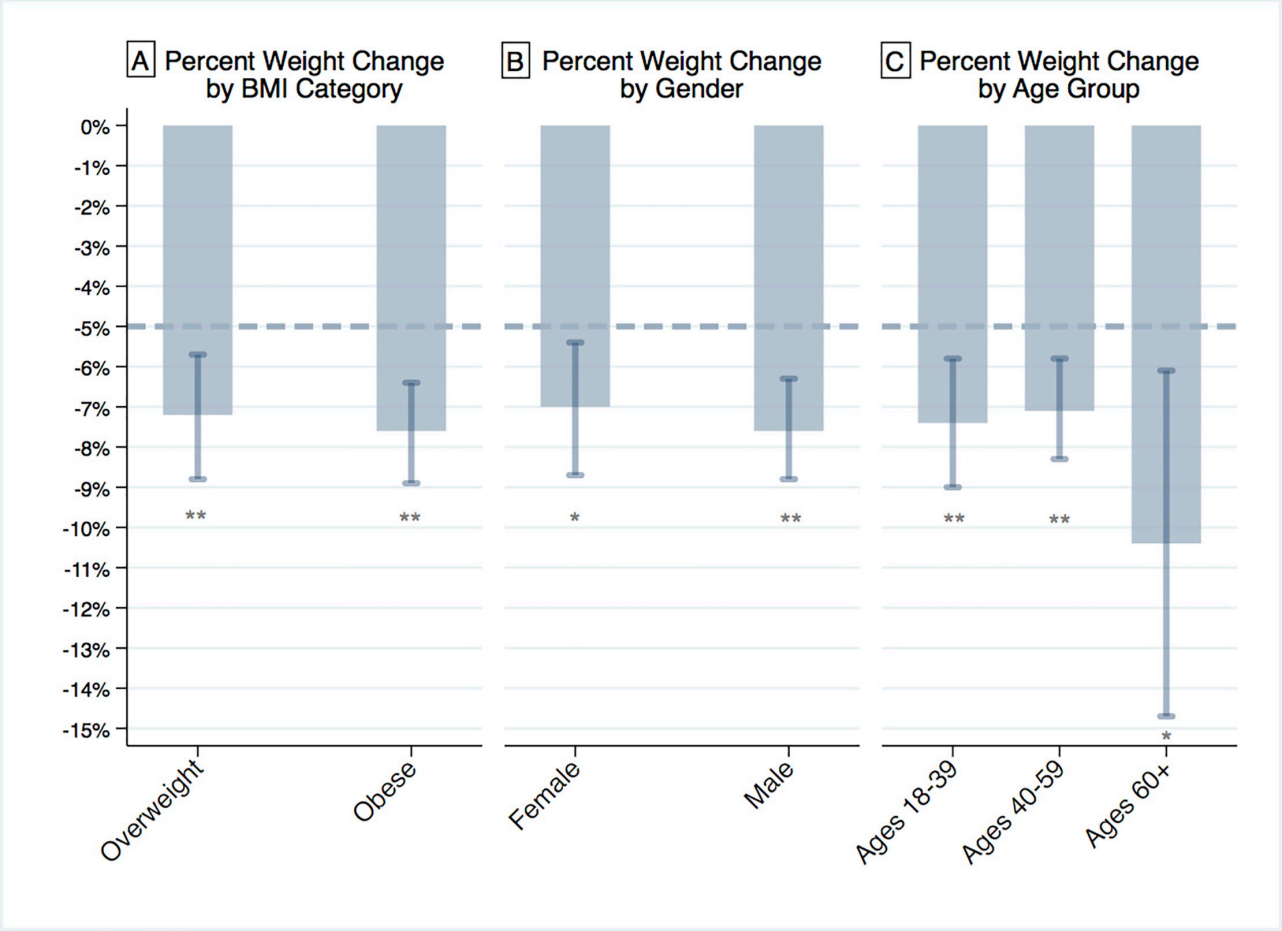
Twelve-month adjusted mean weight loss as a percentage of baseline body weight. A, Mean 12-month weight loss by baseline BMI category. B, Mean 12-month weight loss by gender. C, Mean 12-month weight loss by age group. Error bars represent 95% confidence intervals. *Significantly more weight loss than the 5% benchmark, *P* < .05. **Significantly more weight loss than the 5% benchmark, *P* < .01.

Mean 12-month weight reduction for the overweight group was 7.2% (Table 2), exceeding the aforementioned 5% benchmark by a significant 2.2% (95% CI, 0.7% to 3.8%; *P* < .01; Fig 1A). The obese group's mean weight reduction of 7.6%, similarly, exceeded the 5% benchmark, by 2.6% (95% CI, 1.4% to 3.9%; *P* < .01). Significant heterogeneity was observed across participants in baseline weight (χ2(1) = 85,929, *P* < .01) and rates of change in weight (χ2(1) = 20,586, *P* < .01). Heterogeneity across coaches was significant for baseline weight (χ2(1) = 3.95, *P* < .05), but not for rates of change (χ2(1) = 0.03, *ns*).

**Table 2:** Weight outcomes by baseline BMI category and demographic group.

	Baseline Weight in Pounds^a^ (95% CI)	12-Month Weight in Pounds^a^ (95% CI)	Mean Weight Loss Percentage	Difference from 5% Weight Loss Benchmark (95% CI)	*P* Value^b^
**Group**					
Overweight at Baseline	176.8 (173.0 to 180.6)	164.0 (159.4 to 168.6)	7.2%	2.2% (0.7% to 3.8%)	< .01
Obese at Baseline	231.1 (227.1 to 235.1)	213.4 (208.5 to 218.3)	7.6%	2.6% (1.4% to 3.9%)	< .01
18–39 Years of Age^c^	198.5 (193.6 to 203.5)	183.9 (178.0 to 189.7)	7.4%	2.4% (0.8% to 4.0%)	< .01
40–59 Years of Age^c^	212.3 (207.8 to 216.8)	197.3 (192.1 to 202.5)	7.1%	2.1% (0.8% to 3.3%)	< .01
60+ Years of Age^c^	203.3 (189.5 to 217.1)	182.1 (165.8 to 198.4)	10.4%	5.4% (1.1% to 9.7%)	< .05
Female^c^	186.2 (181.2 to 191.2)	173.1 (167.3 to 179.0)	7.0%	2.0% (0.4% to 3.7%)	< .05
Male^c^	217.7 (213.3 to 222.2)	201.2 (196.1 to 206.4)	7.6%	2.6% (1.3% to 3.8%)	< .01

Abbreviation: BMI, body mass index.

^a^Adjusted marginal means

^b^Comparisons between observed weight changes and the 5% weight loss benchmark.

^c^Adjusted marginal means control for age group and gender, as well as their interactions with time and with each other.

### Weight change across demographic groups

Female and male groups showed mean 12-month weight reductions of 7.0% and 7.6%, respectively (Table 2). This exceeded the 5% benchmark by 2.0% (95% CI, 0.4% to 3.7%; *P* < .05) for females and 2.6% (95% CI, 1.3% to 3.8%; *P* < .01) for males (Fig 1B). Age Group 1 (18–39 years of age) exceeded the 5% weight loss benchmark by 2.4% (95% CI, 0.8% to 4.0%; *P* < .01; Table 2, Fig 1C) while Age Group 2 (40–59 years of age) exceeded the benchmark by 2.1% (95% CI, 0.8% to 3.3%; *P* < .01). Age Group 3 (60+ years of age), finally, exceeded the benchmark by 5.4% (95% CI, 1.1% to 9.7%); *P* < .05).

Weight reduction appeared more pronounced for the oldest group, an unexpected observation. A post hoc contrast was therefore performed, comparing rate of change in weight for the older adult group to the mean rate across the two younger groups. Mean rate of change across the younger groups was -0.28 pounds per week, versus -0.36 pounds per week in the older adult group. The between-groups difference of -0.08 pounds per week (95% CI, -0.23 to 0.09) was not significant.

A significant (*P* < .05) time ✕ gender ✕ age group interaction was also observed. Age Group 1 showed a more negative slope for time than Age Group 2, in female (rate difference = 0.103 pounds per week; 95% CI, -0.01 to 0.22; *P* = .08) but not male (rate difference = -0.06 pounds per week; 95% CI, -0.17 to 0.05; *ns*) participants. Rates of change in weight are presented in Table 3 for each of 6 cells (gender ✕ 3 age groups).

**Table 3:** Rates of change across demographic groups.

		No. (%)	Mean Rate of Change in Weight (Pounds per Week)^a^	95% CI	*P* Value
**Gender**	**Age Group**				
Female					
	Group 1: 18–39 Years of Age	149 (21.8)	-0.318	-0.40 to -0.24	< .01
	Group 2: 40–59 Years of Age	138 (20.2)	-0.215	-0.30 to -0.13	< .01
	Group 3: 60+ Years of Age	14 (2.0)	-0.27	-0.51 to -0.02	< .05
Male					
	Group 1: 18–39 Years of Age	150 (22.0)	-0.26	-0.35 to -0.18	< .01
	Group 2: 40–59 Years of Age	211 (30.9)	-0.32	-0.39 to -0.26	< .01
	Group 3: 60+ Years of Age	21 (3.1)	-0.45	-0.65 to -0.24	< .01

A significant (*P* < .05) time ✕ gender ✕ age group interaction was observed. Age Group 1 showed a more negative rate of change than Age Group 2, in female (rate difference = 0.103 pounds per week; 95% CI, -0.01 to 0.22; *P* = .08) but not male (rate difference = -0.06 pounds per week; 95% CI, -0.17 to 0.05; *ns*) participants.

^a^Average marginal effects by gender and age group.

### Weight reductions following the intensive phase

At 6-months (conclusion of the intensive phase), weight reductions were 2.8% (95% CI, 2.1% to 3.6%; *P* < .01) and 3.2% (95% CI, 2.6% to 3.8%; *P* < .01), for overweight and obese groups respectively.

### Associations between engagement and weight outcomes

Means and standard deviations for each engagement variable are provided in Table 4. Sending more messages to health coaches, manually tracking health metrics more often, and opening more program lessons were associated with more weight loss, to marginally significant degrees. No other engagement variables showed significant or marginally significant associations with weight changes. Relative to participants at the 25th percentile on message sending frequency, those at the 75th percentile lost 2.17 pounds more (95% CI, -0.33 to 4.66; *P* = 0.09) on average. Moving from the 25th to the 75th percentile on manual metric tracking frequency was associated with 0.97 pounds (95% CI, -0.18 to 2.12; *P* = 0.098) more reduction in 12-month weight. Those at the 75th percentile for number of program lessons opened lost 6.71 pounds more (95% CI, -1.15 to 14.57; *P* = 0.09), on average, than those at the 25th percentile. Finally, in contrast to prior findings [25], more frequent weigh-ins were marginally associated with attenuation of weight loss. Moving from 25th to 75th percentiles on weigh-in frequency was associated with 0.87 pounds (95% CI, -0.02 to 1.76; *P* = .06) less weight loss at 12 months.

**Table 4:** Means (SDs) of engagement variables.

Engagement Variable	Events per Week Mean (SD)
Health coaching consults^a^	0.25 (0.23)
Text messages sent to health coach	1.15 (1.60)
Manual health metric tracking	1.74 (4.02)
Automated health metric tracking	14.48 (28.14)
Boolean metric tracking^b^	0.05 (0.25)
Lesson opens^a^	0.24 (0.24)
Lessons completions	0.17 (0.18)
App opens	8.01 (9.35)
Views of personal health analytics	0.76 (1.31)
Content card^c^ opens	0.15 (0.30)

All variables were averaged within participants, across program weeks.

^a^Protocol specified coaching sessions far more frequently during the initial, 24-week intensive phase; session frequency decreased to a monthly cadence subsequently. Session counts were of greatest interest during the intensive phase and were therefore included in analyses. Similarly, counts of lesson opens and completions were analyzed from the initial, 24-week intensive program phase.

^b^These included custom goals coaches set with participants, such as walking for 20 minutes per day.

^c^Brief pieces of content on various health topics, sent at the coach's discretion.

### Attrition analysis

Fifty-two percent of participants had 12-month weight data available. An analysis was performed to investigate the risk that participants who attrit differ systematically from those who do not. If, for example, motivated participants are retained for longer, and they lose more weight, this could bias study results. To investigate this possibility, mean 9-month weights were compared across two groups: participants who had weight observations available at 12+ months and those who did not.

If attrition bias were exaggerating effect size, we would expect 9-month weights of those who had 12+ month observations available to be lower, on average. However, the mean 9-month weight for those who had 12+ month data available was a non-significant 1.22 pounds greater (95% CI, -7.60 to 10.04; *P* = .79) than mean 9-month weight of those who lacked 12+ month weight observations. This finding fails to support the hypothesis that attrition bias increased effect size estimates pertaining to 12-month weight results.

### Sensitivity analysis

Sixty-five percent of weight observations were imported from connected devices, while 35% were entered manually. A sensitivity analysis showed 0.28 pounds (95% CI, 0.53 to 0.03; *P* < .05) more weight loss at 12 months after excluding manually-entered (and thus less reliable) weight observations. This difference represents approximately one-tenth of 1% of the average baseline weight. Manual weights were retained in final analyses for two reasons. First, by no means did retaining manual weights change results to a degree that is *clinically* significant, even if it is statistically significant in this large sample. Second, retaining manual weights caused a slight decrease in effect size, and is therefore slightly conservative.

## Discussion

In this observational study of 683 overweight or obese adults, participation in a digital health intervention with one-on-one health coaching was associated with clinically and statistically significant weight reductions at 12 months. For all BMI categories, age groups, and genders, mean weight loss significantly exceeded the 5% weight reduction benchmark that is often targeted for in-person weight management interventions.

### Key implications

With U.S. obesity and overweight prevalences reaching 40% and 32% respectively [1,2], the importance of scalability in weight management interventions continues to increase. The present results show one example of a scalable weight management intervention with preliminary outcomes comparable to those targeted by in-person interventions that are far less scalable. With further development and research, digitally-delivered weight management interventions may be critical for mitigating the high prevalence of overweight and obesity in the United States, as well as other countries.

### Weight loss patterns across demographics: A candidate explanation

One candidate explanation for the time ✕ gender ✕ age interaction (see Table 3) is that menopause attenuated weight loss for some women in Age Group 2 (40–59 years old); this would be expected to occur rarely in Age Group 1 (18–39 years old). This explanation is consistent with prior evidence [26], and with a marginally significant difference (rate difference = 0.103 pounds per week; 95% CI, -0.01 to 0.22; *P* = .08) between the rates of change observed in Age Groups 1 and 2, for female participants. The corresponding contrast for males, across Age Groups 1 and 2, was not significant (rate difference = -0.06 pounds per week; 95% CI, -0.17 to 0.05; *ns*). This explanation is speculative and these contrasts are post hoc.

### Heterogeneities in rates of change and baseline weights

The coach-level random effect for rate of change in weight was not significant; this would not support a hypothesis of heterogeneity across coaches in weight loss success. In contrast, significant heterogeneity was observed within coaches, across participants, in rates of change for weight. These findings suggest that coaching effectiveness may be consistent across coaches, though the average coach works with participants who achieve varying levels of weight loss success. Not surprisingly, random intercepts were significant at both coach and participant levels. This suggests simply that baseline weights varied significantly, both across and within coaches.

### Limitations

Key study limitations include a retrospective, observational design that precludes causal conclusions. Heterogeneity in weight measurement method is also a limitation, though a sensitivity analysis showed that this did not impact conclusions meaningfully.

Of those who completed in-app program enrollment forms, 36.5% reported an initial weight but did not engage subsequently, and were therefore excluded. This is an important study limitation. It is possible that those excluded differ systematically from those who were included, limiting generalizability.

The enrollment process for this program was designed to minimize barriers to participation. Prospective participants enter contact and demographic information, date of birth, height, weight, and self-reported health conditions, and are then considered enrolled. Those who have limited motivation for health behavior change may be more likely to enroll in this program, relative to programs with more extensive enrollment procedures. Early dropout is common in chronic disease management programs. This is evidenced, for example, by the CDC's policy of including only those Diabetes Prevention Program participants who completed three or more sessions in program evaluations [21]. Alternate approaches we could have utilized include implementing more arduous enrollment protocols and limiting analyses to those who meet a participation threshold. We chose, however, to minimize participation barriers, and to include as much data as possible in analyses. In any case, like many studies of digital health interventions, the present study may have limited generalizability to adults with low motivation for health behavior change.

As is usually the case for studies of digital health interventions, attrition bias is a concern. An analysis was performed to assess the likelihood that those who attrit differ systematically from those who do not. Results showed that data do not support the hypothesis of attrition bias. Nonetheless, we cannot rule it out. It remains possible that participants who attrit before 12 months are systematically different, and that such participants showed less weight loss. While the aforementioned analysis fails to support this hypothesis, if it is true, then study results would be biased toward effect size overestimation. On the other hand, if participants who disengaged before 12 months did so because they reached weight loss goals faster and no longer perceived a need for the intervention, then results reported herein would underestimate weight loss. In any case, the possibility that attrition biased results and decreased generalizability remains an important limitation.

### Future directions

Future research is needed to investigate outcome durability, explore the impact on costs, and confirm findings with a randomized design that includes in-person and usual care controls. Future efforts should of course include aggressive follow-up to minimize attrition. Studies are also needed to test innovative digital therapeutic approaches, leveraging advanced behavioral and data science, to target results that are both more scalable and more efficacious than in-person programs. Additional research is needed, moreover, to elucidate which program engagement variables predict weight outcomes for which participants. This may allow us to tailor program protocols, focus on intervention components that are most likely to drive weight reductions for each unique participant, and increase program efficacy.

Future studies are also needed, with a larger sample of older adults, to investigate whether they may benefit more than younger adult participants. More broadly, additional work is needed to optimize program adaptations for the unique needs of different demographic groups, especially minority populations that show elevated prevalence of overweight and obesity [2].

## Conclusions

Despite limitations, this study may have important implications. Current guidelines [8,9] recommend in-person weight management interventions as preferable to those delivered remotely. The present findings, however, suggest that some digital health interventions may achieve weight reductions that are at least comparable to outcomes observed for robust, in-person programs. Although more research is needed, this preliminary evidence appears promising. With additional development and research, digital health interventions for obesity could play a vital role in mitigating the current epidemic of obesity.
